# Cognitive Reserve and Its Relationship With Memory Changes: An Analysis of the Survey of Health, Aging, and Retirement in Europe (SHARE)

**DOI:** 10.1002/gps.70190

**Published:** 2026-01-06

**Authors:** Juan C. Melendez, Luis Carlos Venegas, Claire P. de la Fuente

**Affiliations:** ^1^ Department of Developmental Psychology Faculty of Psychology University of Valencia Valencia Spain; ^2^ Hospital Universitario Mayor ‐ Méderi Bogotá Colombia; ^3^ Instituto Rosarista para el Estudio del Envejecimiento y la Longevidad Universidad del Rosario Bogotá Colombia

**Keywords:** aging, cognitive reserve proxies, dementia risk factors, depression, episodic memory, SHARE study

## Abstract

**Objectives:**

To examine the longitudinal association between cognitive reserve (CR)‐related proxies and episodic memory in older adults, and to explore the role of sociodemographic and clinical risk factors.

**Methods:**

Data were drawn from 2279 participants of the Survey of Health, Aging and Retirement in Europe (SHARE), with baseline in wave 5 (2013) and follow‐up in wave 9 (2021–2022). A CR‐proxy score was constructed using education, occupation, physical activity, social engagement, and loneliness. Logistic regression models were used to predict immediate and delayed recall performance at follow‐up, adjusting for age, sex, depression, vascular risk factors, and sensory impairments.

**Results:**

Higher levels of CR‐related proxies significantly reduced the odds of impairment in both immediate recall (OR = 0.55, *p* < 0.001) and delayed recall (OR = 0.46, *p* < 0.001). Age was associated with poorer memory outcomes, while women showed better performance in delayed recall being female predicted lower odds of preserved delayed recall. Depression was significantly related to poorer immediate recall, but other health conditions and sensory factors were not significant predictors.

**Conclusions:**

CR‐related proxies were strong predictors of memory performance over the 9‐year period, particularly for delayed recall. These findings reflect sociobehavioural influences associated with CR development, rather than direct evidence of CR as a neurofunctional mechanism. Promoting cognitively, socially and physically enriching activities, together with addressing depression, may help preserve memory function in aging populations.

## Key Points

- Higher levels of CR‐related proxies were associated with lower risk of memory decline.
- The predictive effect of CR‐related proxies was strongest for delayed recall.
- Depression was the only modifiable risk factor significantly associated with poorer immediate recall.
- Findings support promoting lifelong education, physical activity, and social engagement as CR‐related experiences that contribute to cognitive resilience.

## 1 Introduction

Cognitive aging is a heterogeneous process shaped by biological, psychological, and environmental factors. A central explanatory construct is cognitive reserve (CR), defined as the capacity of the brain to sustain cognitive performance through efficient and flexible recruitment of neural networks, even in the presence of age‐related pathology [1, 2, 3]. According to recent consensus frameworks, CR cannot be measured directly; instead, it is inferred from patterns of performance or from life experiences that are thought to contribute to its development [3, 4, 5, 6]. Such experiences commonly indexed through education, occupational complexity, or engagement in physical and social activities, should therefore be considered proxies associated with CR rather than direct measurements of the construct. Episodic memory is highly vulnerable to age‐related decline [7, 8]. However, the course of this decline is heterogeneous, and cognitive reserve (CR) has been proposed as a key explaining mechanism underlying such variability. Empirical studies consistently show that individuals with life experiences associated with higher CR proxies tend to perform better and maintain memory function over time, even in the presence of brain pathology, likely through more efficient and flexible neural network recruitment [9, 10, 11, 12].

In addition to CR‐related proxies, several modifiable risk factors influence cognitive aging. Vascular conditions, depression, sensory impairments, and lifestyle‐related factors have all been identified as contributors to cognitive decline. The Lancet Commission on dementia prevention, intervention, and care emphasizes that both health‐related conditions and sociobehavioural factors such as education, physical activity, and social engagement, that play a critical role in shaping cognitive trajectories [13]. This framework aligns with the CR literature, as many of these sociobehavioural experiences are also considered proxies associated with CR development, yet the extent to which they interact prospectively with memory performance remains insufficiently understood.

The aim of this study was to analyze the longitudinal association between sociobehavioural factors commonly used as proxies associated with cognitive reserve (CR) and episodic memory performance, both immediate and delayed, in a large cohort of older European adults from the SHARE survey. Using a 9‐year follow‐up design, we also evaluated the independent and combined effects of sociodemographic and clinical risk factors identified in the CR and Lancet Commission frameworks. We hypothesized that higher levels across CR‐related proxies would be associated with better memory performance, independently of health‐related risk factors, thereby contributing evidence to guide public health interventions.

## 2 Materials and Methods

### 2.1 Study Design and Participants

This is a longitudinal cohort study based on secondary analysis of the Survey of Health, Aging and Retirement in Europe (SHARE), a multidisciplinary panel survey initiated in 2004 that collects data biennially from adults aged 50 and older across multiple European countries. SHARE uses a probability‐based sampling design and gathers detailed information on health, socioeconomic status, and social networks. Ethical approval for SHARE was obtained from the Ethics Council of the Max Planck Society, and all participants provided written informed consent at enrollment.

For the present study, data from wave 5 (2013) were used as baseline and wave 9 (2021–2022) as follow‐up, resulting in an observation period of approximately nine years. Participants were eligible if they (a) had complete data in both waves, (b) were aged 65 years or older at baseline, and (c) reported being retired. Exclusion criteria included self‐reported diagnosis of dementia, stroke, or Parkinson's disease, and any limitations in instrumental activities of daily living (IADLs), assessed with SHARE items comparable to the Lawton and Brody scale. To maximize comparability, listwise deletion was applied and only complete cases were analyzed.

Although the study uses a longitudinal structure with baseline predictors (Wave 5) and cognitive outcomes at 9‐year follow‐up (Wave 9), the objective was to examine whether baseline sociobehavioural and health‐related factors predict later memory performance rather than to model intra‐individual cognitive trajectories. Consequently, analyses focus on follow‐up cognitive status instead of change metrics or longitudinal trajectory modeling.

### 2.2 Measures

Following current conceptual frameworks [3, 6], cognitive reserve (CR) cannot be measured directly; instead, it is inferred from life experiences that are theorized to contribute to its development. In the present study, we therefore operationalized a set of sociobehavioural proxies associated with CR using five dichotomized variables from SHARE Wave 5: (i) educational attainment (> 12 years = high), (ii) last occupation with high cognitive complexity (managerial, scientific, technical, administrative, skilled manual, or military roles = high), (iii) weekly participation in structured social activities (yes/no), (iv) weekly engagement in vigorous physical exercise (yes/no), and (v) absence of loneliness, defined as a score ≤ 3 on the SHARE adaptation of the UCLA Loneliness Scale (yes/no). Because these indicators represent heterogeneous experiences rather than reflective manifestations of a single latent construct, exploratory factor analysis was used only as a data‐driven approach to derive empirical weights for a composite proxy score, without assuming that the extracted component reflects CR as a unitary factor. A principal‐component solution was retained, and factor loadings from this component were used to compute a weighted continuous CR‐proxy score, which served as the sociobehavioural predictor in subsequent analyses.

Health‐related variables. In accordance with the Lancet Commission on dementia prevention, intervention, and care, we included several modifiable risk factors from SHARE wave 5. All variables were coded dichotomously (0 = absent, 1 = present). Hypertension, diabetes, and dyslipidemia were based on self‐reported physician diagnoses. Vision and hearing impairments were operationalized as the reported use of corrective lenses and hearing aids, respectively. Depression was assessed with the EURO‐D scale and coded as present if the EURO‐D categorical variable indicated depression.

Sociodemographic covariates. Age (continuous, in years) and sex were included as covariates. Sex was coded as a binary variable, with males as the reference category.

Memory outcomes. Episodic memory was assessed in SHARE wave 9 using a standardized 10‐word list recall test. In the immediate recall task, participants repeated as many words as possible directly after auditory presentation. In the delayed recall task, the same list was recalled after a five‐minute distractor activity. Each correct word was scored as one point, yielding a maximum of 10 points per task. For analytic purposes, scores were dichotomized separately for immediate and delayed recall: participants scoring ≤ 1 standard deviation below the sample mean were classified as having low performance, while those scoring above this threshold were classified as normal performers.

### 2.3 Statistical Analysis

An exploratory factor analysis with principal component extraction was applied to derive empirical weights for the CR‐proxy score; this procedure was not intended to validate a latent CR construct but to generate a data‐driven weighting scheme for the heterogeneous sociobehavioural proxies associated with CR. We used Wave 9 memory performance as the dependent variable because the aim of the study was to test whether baseline factors predict subsequent cognitive status.

Sampling adequacy was evaluated with the Kaiser‐Meyer‐Olkin measure and Bartlett's test of sphericity. The resulting factor loadings were used to compute the weighted CR‐proxy score.

Two separate binary logistic regression models were estimated to examine predictors of immediate and delayed recall performance. Independent variables included the CR‐proxy score (continuous), age (continuous), sex (male = reference), and all health‐related covariates (coded dichotomously). Model fit was assessed with the chi‐square statistic, Nagelkerke's *R* ^2^, and the Hosmer‐Lemeshow goodness‐of‐fit test. For each predictor, regression coefficients (*B*), standard errors, Wald statistics, odds ratios (Exp(*B*)), and 95% confidence intervals were reported.

All analyses were conducted using IBM SPSS Statistics version 25.0. Assumptions of logistic regression were checked, and all tests were two‐tailed with *p* < 0.05 considered statistically significant.

## 3 Results

### 3.1 Baseline Characteristics

From an initial sample of 66,038 individuals, a total of 2279 participants met all inclusion criteria and were retained for analysis. At baseline, participants had a mean age of 71.11 years (SD = 4.81), with women comprising 48.3% of the sample. The average educational attainment was 11.73 years (SD = 3.96). Most participants were married and cohabiting with their spouse (see Table 1).

**TABLE 1:** Baseline characteristics and differences between high and low cognitive reserve index (P_75_) (*n* = 2279).

Variable	Low CR (*n* = 1598)	High CR (*n* = 681)	*p*‐value
Age, mean (SD)	71.5 (5.0)	70.1 (4.3)	< 0.001
Female, *n* (%)	818 (51%)	283 (42%)	< 0.001
Visual impairment, *n* (%)	1330 (83%)	579 (85%)	0.30
Hearing impairment, *n* (%)	151 (9.4%)	73 (11%)	0.40
Hypertension, *n* (%)	717 (45%)	283 (42%)	0.14
Diabetes, *n* (%)	389 (24%)	172 (25%)	0.60
Dyslipidemia, *n* (%)	201 (13%)	63 (9.3%)	0.02
Depression, *n* (%)	272 (17%)	74 (11%)	< 0.001
Baseline immediate recall, mean (SD)	5 (2)	6 (2)	< 0.001
Baseline delayed recall, mean (SD)	4 (2)	5 (2)	< 0.001
Follow‐up immediate recall, mean (SD)	5 (2)	5 (2)	< 0.001
Follow‐up delayed recall, mean (SD)	3 (2)	4 (2)	< 0.001

### 3.2 Exploratory Factor Analysis of the CR‐Proxy Score

An exploratory factor analysis was conducted on the five variables selected to construct the CR‐proxy score. The Kaiser‐Meyer‐Olkin measure of sampling adequacy yielded a moderate value of 0.560, and Bartlett's test of sphericity was statistically significant (*p* < 0.001), indicating that the data were suitable for principal component extraction A single principal component accounted for 28.9% of the variance and its loadings: educational attainment (0.722), occupational complexity (0.688), social participation (0.511), physical activity (0.355), and absence of loneliness (0.256), were used to compute the weighted CR‐proxy score. The mean CR‐proxy score was 1.51 (SD = 0.70). Using the 75th percentile cut‐off (score = 2.02), 681 participants (29.9%) were classified as having high CR‐proxy levels, whereas 1598 participants (70.1%) scored below this threshold. Baseline characteristics and differences between subjects with high and low CR were reported in Table 1.

### 3.3 Dichotomization of Immediate and Delayed Memory Scores

Immediate and delayed memory scores were obtained from SHARE wave 9. To create binary outcome variables suitable for logistic regression, a cut‐off of one standard deviation below the mean was applied. For immediate memory, the mean score was 4.90 (SD = 1.61), resulting in a threshold of 3.28. Participants scoring 3.28 or lower were categorized as having low immediate memory, while those scoring above this threshold were considered to have normal immediate memory. Based on this criterion, 383 participants (16.8%) were classified as having low immediate memory, and 1896 participants (83.2%) as having normal performance.

In the case of delayed memory, the mean score was 3.49 (SD = 2.07), with a threshold of 1.41 used to identify low performance. Participants scoring 1.41 or lower were classified as having low delayed memory, and those above this cut‐off as having normal delayed memory. A total of 67 participants (2.9%) were identified as having low delayed memory, while 2212 participants (97.1%) were categorized as having normal performance.

### 3.4 Binary Logistic Regression Model for Immediate Memory

Table 2 presents the results of the logistic regression analysis examining predictors of immediate recall performance. Higher CR‐proxy levels were significantly associated with lower odds of impairment (*B* = −0.606, Exp(*B*) = 0.546, *p* < 0.001, 95% CI [0.460, 0.647]). Older age increased the likelihood of poorer immediate recall (*B* = 0.113, Exp(*B*) = 1.120, *p* < 0.001, 95% CI [1.095, 1.146]). Being female was associated with substantially lower odds of low performance (*B* = −0.529, Exp(*B*) = 0.124, *p* < 0.001, 95% CI [0.462, 0.751]), indicating better immediate memory performance compared with men. Additionally, depressive symptoms significantly predicted worse performance (*B* = 0.392, Exp(*B*) = 1.480, *p* = 0.012, 95% CI [1.092, 2.007]). Other variables, including visual and hearing impairment, hypertension, diabetes, and dyslipidemia, were not significant predictors. The model demonstrated acceptable goodness of fit, as indicated by a non‐significant Hosmer‐Lemeshow test (χ^2^(8) = 8.77, *p* = 0.362), and accounted for 14.1% of the variance in immediate recall performance (Nagelkerke's *R* ^2^ = 0.141).

**TABLE 2:** Regression analysis for immediate recall.

Variable	B	Exp(B)	Wald	Sig.	CI 95% inf.	CI 95% sup.
Cognitive reserve	−0.606	0.546	48.284	0.000	0.460	0.647
Age	0.113	1.120	94.366	0.000	1.095	1.146
Female	−0.529	0.124	0.589	0.000	0.462	0.751
Visual impairment	−0.190	0.827	1.524	0.217	0.611	1.118
Hearing impairment	0.231	1.260	1.628	0.202	0.883	1.798
Hypertension	0.023	1.023	0.035	0.852	0.804	1.302
Diabetes	0.094	1.099	0.455	0.500	0.835	1.446
Dyslipidemia	−0.084	0.919	0.209	0.647	0.642	1.318
Depression	0.392	1.480	6.385	0.012	1.092	2.007

*Note:* Hosmer‐Lemeshow test, χ^2^(8) = 8.77, *p* = 0.362; Nagelkerke's *R* ^2^ = 0.141.

### 3.5 Binary Logistic Regression Model for Delayed Memory

As shown in Table 3, higher CR‐proxy levels emerged as a significant protective factor for delayed recall, with higher levels associated with a notably lower likelihood of impairment (*B* = −0.775, Exp(*B*) = 0.461, *p* < 0.001, 95% CI [0.319, 0.666]). Advancing age was associated with an increased risk (*B* = 0.091, Exp(*B*) = 1.095, *p* < 0.001, 95% CI [1.046, 1.147]). Additionally, being female was associated with lower odds of low delayed performance (*B* = −0.584, Exp(*B*) = 0.558, *p* = 0.028, 95% CI [0.331, 0.939]), again reflecting better memory outcomes among women. Other health‐related variables, including sensory impairments, cardiovascular and metabolic conditions, and depressive symptoms, did not reach statistical significance in this model. Overall, the model showed excellent calibration (Hosmer‐Lemeshow χ^2^(8) = 1.37, *p* = 0.995) and accounted for 12.1% of the variance in delayed recall (Nagelkerke's *R* ^2^ = 0.121).

**TABLE 3:** Regression analysis for delayed recall.

Variable	B	Exp(B)	Wald	Sig.	CI 95% inf.	CI 95% sup.
Cognitive reserve	−0.775	0.461	16.952	0.000	0.319	0.666
Age	0.091	1.095	15.109	0.000	1.046	1.147
Female	−0.584	0.558	4.824	0.028	0.331	0.939
Visual impairment	−0.411	0.663	1.875	0.171	0.368	1.194
Hearing impairment	−0.178	0.837	0.179	0.672	0.367	1.908
Hypertension	0.172	1.188	0.437	0.508	0.713	1.980
Diabetes	0.088	1.092	0.086	0.770	0.607	1.964
Dyslipidemia	−0.534	0.586	1.436	0.231	0.245	1.404
Depression	−0.014	0.986	0.002	0.967	0.499	1.947

*Note:* Hosmer‐Lemeshow test, χ^2^(8) = 1.37, *p* = 0.995; Nagelkerke's *R* ^2^ = 0.121.

## 4 Discussion

Our findings indicate that higher levels of CR‐related proxies are a robust predictor of both immediate and delayed memory performance, even after adjusting for a broad range of risk factors. This is consistent with previous research showing that life experiences commonly used as proxies for CR are associated with better cognitive outcomes. In line with contemporary CR frameworks (Stern et al., 2020, 2023), these associations should be interpreted as reflecting the predictive value of CR‐related proxies rather than direct evidence of CR as a neurofunctional mechanism, given that demonstrating CR requires testing moderation between brain pathology and cognition, a test not feasible here due to the lack of neurobiological measures. These proxies represent formative exposures linked to the development of CR, but do not constitute a direct measurement of the underlying CR mechanism [3, 6]. Notably, the effect of CR‐proxies was more pronounced in delayed memory tasks, suggesting a specific benefit in processes that involve memory consolidation. These findings underscore the importance of targeting CR‐building activities across the lifespan to promote resilience against age‐related memory deterioration.

The CR‐proxy score used in this study was constructed through exploratory factor analysis and included established proxies: educational attainment, occupational complexity, physical activity, social engagement, and loneliness. These components have been previously identified as meaningful contributors to CR [14, 15, 16]. Although loneliness showed the weakest factor loading, its inclusion is theoretically and empirically justified given its known negative impact on cognitive performance, particularly on memory and processing speed [17].

Our findings align with literature showing strong links between CR‐related proxies and episodic memory performance [8, 18, 19]. It is important to acknowledge, in line with contemporary frameworks [3, 6], that these proxies do not constitute a direct measure of CR but represent life experiences associated with its development. Accordingly, the present study fits within the scenario in which CR is inferred indirectly from sociobehavioural indicators in the absence of neurobiological measures. Within this context, research by Sandrini et al. supports the idea that individuals with higher CR‐related proxies not only perform better spontaneously but also respond more positively to neurocognitive interventions, such as transcranial direct current stimulation [11].

The stronger association between CR‐related proxies and delayed memory may be attributed to processes typically associated with CR, such as enhanced neural efficiency and compensatory recruitment. The delayed memory task, which requires sustained encoding and retrieval over time, benefits from distributed and efficient brain network engagement. In aging individuals, CR facilitates the recruitment of alternative neural circuits to compensate for age‐related neurofunctional decline [2, 12]. This compensatory model helps explain why individuals with higher CR may perform better in delayed recall tasks.

Age, as expected, was negatively associated with both types of memory performance. This supports previous findings [6, 20, 21] and reflects multiple converging factors, including synaptic deterioration, reduced processing speed, and executive dysfunction. The loss of inhibitory control and reduced flexibility with age likely interfere with both encoding and retrieval processes, thus lowering memory performance [22, 23]. Additionally, self‐perception of cognitive decline and increased internal distractibility may further exacerbate memory impairments in older adults [24].

Sex was another significant predictor, with women outperforming men in both memory domains. This finding is consistent with the literature showing female advantages in verbal and episodic memory [25, 26]. This may stem from both biological (e.g., larger hippocampal volume, estrogen‐related benefits) and cognitive‐behavioral mechanisms (e.g., better use of verbal encoding strategies) [27, 28]. However, such differences should be interpreted as domain‐specific advantages rather than generalized cognitive superiority.

Among the modifiable risk factors examined, depression emerged as the only variable significantly associated with poorer immediate memory. This finding underscores the clinical relevance of depressive symptoms in older adults, highlighting the importance of systematic screening and treatment of depression within geriatric psychiatry to help preserve memory performance. This supports prior evidence suggesting that depressive symptoms disproportionately affect attention, encoding, and working memory processes [29]. In contrast, delayed memory, requiring longer‐term consolidation, appeared less vulnerable to the immediate effects of depressive mood [30]. Neurobiologically, the link may be mediated through the prefrontal cortex, whose dysfunction in depression may impair executive control more than hippocampal‐mediated consolidation processes [31].

Corrective lens use was not associated with poorer memory performance. This is consistent with findings that suggest the correction of visual impairments may mitigate cognitive decline risk, potentially by reducing sensory‐cognitive load and enabling greater environmental engagement [32, 33]. Similarly, no negative effects of hearing aid use were observed, aligning with studies suggesting these devices buffer against cognitive decline by reducing sensory deprivation and promoting social interaction [34].

No significant associations were found between memory performance and clinical conditions such as hypertension, diabetes, or dyslipidemia. These findings diverge from well‐documented associations in the literature [12, 35]. One plausible explanation is that many participants were receiving effective medical treatment for these conditions, potentially mitigating their cognitive impacts. Indeed, studies have shown that antihypertensives [36], statins [37], and glucose‐lowering agents [38] may offer neuroprotective effects, especially when adherence is high and treatment begins early. It is also possible that the effect of age captured the cumulative vascular burden, reducing the independent effect of each factor in the multivariate models.

Clinically, these results have key implications for designing public health strategies aimed at supporting cognitive health in aging populations. Interventions aimed at increasing CR‐related experiences, such as lifelong education, physical exercise, and community engagement, should be prioritized. These activities are not only associated with better cognitive outcomes but are modifiable and implementable at the population level [39, 40]. Programs promoting cognitive stimulation, physical activity, and social connection should be embedded within age‐friendly community planning and public policy.

The study also highlights the need for early detection and treatment of depressive symptoms in older adults, given their specific detrimental effect on memory. Although sensory loss and vascular conditions did not show significant associations in this study, their known associations with dementia and cerebrovascular health support ongoing clinical vigilance and intervention.

Importantly, findings related to age and sex emphasize the need for tailored interventions. Prevention should begin well before the onset of cognitive symptoms and should consider individual differences in vulnerability and resilience. Targeted strategies based on sex may improve effectiveness, particularly when focused on strengths in verbal memory for women and compensatory strategies for men.

Several strengths enhance the validity of this study. First, the CR‐proxy score was built using a multidimensional approach grounded in theoretical and empirical literature. Factor analysis enabled weighting of components based on their statistical contribution, resulting in a more nuanced measure than simple additive scoring. Second, the use of data from SHARE ensures high external validity due to its large, representative, and diverse European sample. Third, the longitudinal design, with a follow‐up period of nearly a decade, strengthens the directionality of the observed associations.

Nonetheless, limitations must be acknowledged. The strict inclusion criteria may have led to a sample that is healthier and more functionally independent than the general older adult population. As a result, findings may not generalize to frail or clinically complex individuals. Importantly, although grounded in empirical proxies, our measure still does not capture CR directly, as no neurobiological indicators were available. This is a recognized limitation in studies based on large epidemiological datasets and should be considered when interpreting the findings. Moreover, the exclusive use of immediate and delayed memory as cognitive outcomes limits generalizability to other cognitive domains such as executive functioning or processing speed. Although the design includes two assessment waves separated by 9 years, we did not analyze cognitive trajectories. Our analytic focus was on prospective prediction of follow‐up memory status from baseline factors. Future studies incorporating multiple follow‐up waves or modeling approaches specifically tailored to longitudinal change would help clarify aging trajectories more precisely.

Future research should incorporate neuroimaging and biomarker data to examine whether CR moderates the relationship between brain pathology and cognitive outcomes. Including broader cognitive assessments and expanding the sample to include individuals with mild functional impairments would enhance clinical relevance. Furthermore, longitudinal analyses with multiple follow‐up points could help clarify trajectories and transitions between cognitive states.

In conclusion, this longitudinal cohort study underscores the critical role of CR‐related proxies as predictors of memory performance in aging. In this context, their predictive value reflects sociobehavioural influences on cognitive aging and the impact of life‐course experiences associated with CR development. Promoting CR‐enhancing behaviors across the lifespan, particularly through education, physical activity, and social engagement, emerges as a promising public health priority. Although only depression was directly associated with memory decline in this sample, the effective control of vascular and sensory risk factors remains essential for cognitive health. Taken together, these findings reinforce the need to integrate CR‐enhancing activities and systematic depression management into preventive strategies for cognitive aging.

## Funding

The authors have nothing to report.

## Ethics Statement

Ethical approval for SHARE was obtained from the Ethics Council of the Max Planck Society and previously from the Ethics Committee of the University of Mannheim.

## Consent

The authors have nothing to report.

## Conflicts of Interest

The authors declare no conflicts of interest.

## Permission to Reproduce Material

The authors have nothing to report.

## Data Availability

The data that support the findings of this study are openly available in SHARE – Survey of Health, Aging and Retirement in Europe at http://www.share‐project.org/, reference number Wave 5 → 10.6103/SHARE.w5.800 Wave 9 → 10.6103/SHARE.w9.100.
